# Profiling of H3K27Ac Reveals the Influence of Asthma on the Epigenome of the Airway Epithelium

**DOI:** 10.3389/fgene.2020.585746

**Published:** 2020-12-10

**Authors:** Peter McErlean, Audrey Kelly, Jaideep Dhariwal, Max Kirtland, Julie Watson, Ismael Ranz, Janet Smith, Alka Saxena, David J. Cousins, Antoon Van Oosterhout, Roberto Solari, Michael R. Edwards, Sebastian L. Johnston, Paul Lavender

**Affiliations:** ^1^Peter Gorer Department of Immunobiology, King’s College London, London, United Kingdom; ^2^Asthma UK Centre in Allergic Mechanisms of Asthma, London, United Kingdom; ^3^Airway Disease Infection Section, National Heart and Lung Institute, Imperial College London, London, United Kingdom; ^4^GlaxoSmithKline Allergic Inflammation Discovery Performance Unit, Respiratory Therapy Area, Stevenage, United Kingdom; ^5^Genomics Platform, Biomedical Research Centre at Guy’s and St Thomas’ NHS Foundation Trust, London, United Kingdom; ^6^National Institute for Health Research (NIHR) Respiratory Biomedical Research Unit, Department of Infection, Immunity & Inflammation, Leicester Institute for Lung Health, University of Leicester, Leicester, United Kingdom

**Keywords:** asthma, epigenetics, H3K27ac—Histone 3 lysine 27 acetylation, chromatin, bronchial epithelial cells, lung

## Abstract

**Background:**

Asthma is a chronic airway disease driven by complex genetic–environmental interactions. The role of epigenetic modifications in bronchial epithelial cells (BECs) in asthma is poorly understood.

**Methods:**

We piloted genome-wide profiling of the enhancer-associated histone modification H3K27ac in BECs from people with asthma (*n* = 4) and healthy controls (*n* = 3).

**Results:**

We identified *n* = 4,321 (FDR < 0.05) regions exhibiting differential H3K27ac enrichment between asthma and health, clustering at genes associated predominately with epithelial processes (EMT). We identified initial evidence of asthma-associated Super-Enhancers encompassing genes encoding transcription factors (*TP63*) and enzymes regulating lipid metabolism (*PTGS1*). We integrated published datasets to identify epithelium-specific transcription factors associated with H3K27ac in asthma (*TP73*) and identify initial relationships between asthma-associated changes in H3K27ac and transcriptional profiles. Finally, we investigated the potential of CRISPR-based approaches to functionally evaluate H3K27ac-asthma landscape *in vitro* by identifying guide-RNAs capable of targeting acetylation to asthma DERs and inducing gene expression (*TLR3*).

**Conclusion:**

Our small pilot study validates genome-wide approaches for deciphering epigenetic mechanisms underlying asthma pathogenesis in the airways.

## Background

Asthma is a chronic inflammatory disease of the airways affecting over 230 million people worldwide (WHO, 2014). Driven by complex genetic–environmental interactions, and involving multiple cell types, asthma’s origins, triggers, and clinical presentations are heterogeneous, posing challenges to understanding disease development, molecular components, and the generation of more effective therapies (Wenzel, 2012). To date, only a small percentage of asthma’s genetic association can be explained by SNPs suggesting that epigenetic contribution to disease may be significant.

The airway epithelium of people with asthma is characterized by altered phenotypic and transcriptional characteristics including excessive mucus production, defects in antiviral responses and repair (Kicic et al., 2010; Lambrecht and Hammad, 2012; Edwards et al., 2013), and the predominance of signals associated with type 2 (T2) inflammation (Woodruff et al., 2007; Gordon et al., 2016). However, little is known about the molecular mechanisms that underpin the transcriptional profiles of airway epithelium in asthma.

Studies of epigenomic mechanisms including DNA methylation and histone modifications have determined the epigenomes across a variety of cell types altered in asthma (Yang and Schwartz, 2012; Seumois et al., 2014; Kidd et al., 2015). Chromatin immunoprecipitation coupled with high-throughput sequencing (ChIP-Seq) profiling of histone H3 Lys4 dimethylation (H3K4me2) in CD4^+^ T cells from individuals with asthma has identified putative asthma-associated enhancers (Seumois et al., 2014), supporting the use of genome-wide approaches to identify novel epigenomic mechanisms in asthma. However, while DNA methylation has been broadly investigated (Moheimani et al., 2016; Nicodemus-Johnson et al., 2016; Yang et al., 2017), genome-wide investigations of histone modifications in the airway epithelium in asthma have yet to be undertaken.

Ranking ChIP-Seq signals revealed that regions of the genome exhibit increased enrichment of enhancer-associated histone modifications (e.g., H3 Lys27 acetylation- H3K27ac) in a cell-type and disease-specific manner. These regions, termed “Super-Enhancers” (SEs), exhibit sustained enrichment across several kilobases (kb), encompass “master” transcription factors (TFs) important in cell identity, and are more likely to harbor disease-associated single-nucleotide polymorphisms (SNPs) (Hnisz et al., 2013; Whyte et al., 2013). Super-Enhancers have been identified across numerous cell types and diseases and are of potential interest for therapeutic intervention (Lovén et al., 2013; Peeters et al., 2015; Khan and Zhang, 2016). However, airway epithelial cells and asthma-associated SEs have yet to be described.

In this pilot study, we sought to investigate the influence of asthma on the histone landscape by profiling H3K27ac via ChIP-Seq in bronchial epithelial cells (BECs) from people with asthma and healthy individuals and identifying regions of differential enrichment. By profiling an enhancer-associated histone modification, we additionally sought to identify asthma-associated SEs and identify the TFs associated with altered H3K27ac landscape in asthma. We investigated the relationships between asthma-associated H3K27ac and transcriptional profiles in the airways. Finally, we wished to explore the feasibility of using epigenome editing to functionally address the consequence of altered H3K7ac landscapes *in vitro*.

## Results

### Asthma Influences H3K27ac Enrichment in Airway Epithelial Cells

To investigate the influence of asthma on the epigenome of the airway epithelium, we undertook genome-wide profiling of H3K27ac via ChIP-Seq in BECs from healthy controls (*n* = 3) and adults with asthma (*n* = 4, Supplementary Table S1). We identified regions exhibiting the greatest H3K27ac enrichment for each volunteer (peaks, Supplementary Table S2) and then by comparing enrichment across a consensus peak set identified *n* = 4,321 differentially enriched regions (DERs, FDR < 0.05, DiffBind) between asthma and healthy BECs (Supplementary Table S3).

**FIGURE 1:**
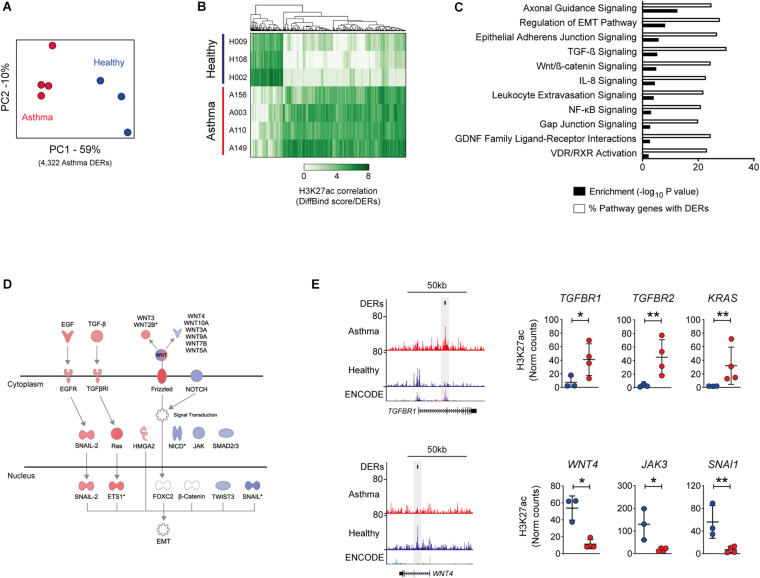
Influence of asthma on the epigenome of the airway epithelium. **(A)** Principal component analysis of H3K27ac across differentially enriched regions (DERs) identified by DiffBind. Clustering identifies variation both between and within healthy (blue) and asthma (red) BECs. **(B)** Heatmap depicting volunteer correlation across asthma DERs and the presence of gain and loss of H3K27ac in asthma BECs. **(C)** DER-associated genes are enriched in pathways associated with epithelial biology and asthma pathophysiology (–log_10_ *P-*values, black bars). Pathway analysis also revealed that up to 30% of genes within the same pathway exhibited differential H3K27ac in asthma (% of pathway genes with DERs, white bars). **(D)** Condensed versions of the epithelial-to-mesenchymal transition (EMT) pathway depicting how numerous components are within the same pathway gain (red) and lose (blue) H3K27ac in asthma BECs. **(E)** Genome tracks depicting H3K27ac enrichment in BECs across example gain (*TGFBR1*) and loss asthma DERs (*WNT4*). Data tracks from bottom to top; genes (RefSeq annotation), layered H3K27ac from ENCODE, merged data from healthy (*n* = 3, blue) and asthma BECs (*n* = 4, red) and Asthma DERs (black boxes). Dot plots depict enrichment across volunteers at select genes with gain and loss DERs (*P < 0.05, **P < 0.01, DiffBind). Asthma = red, healthy = blue.

PCA confirmed that the greatest variation in DERs was observed between study groups, indicating that despite limited *ex vivo* culture, differences in the epigenome were present in BECs (Figure 1A). Asthma DERs encompassed regions with a gain (*n* = 3,061) or loss (*n* = 1,260) in H3K27ac enrichment (Figure 1B) and occurred predominately at distal intergenic and promoter/intronic sites, respectively (Supplementary Figure S1A).

Because our asthmatic volunteers were on inhaled corticosteroids (ICS), we investigated if asthma DERs could be the result of treatment-induced glucocorticoid receptor (GR) binding. We observed 2.3% of asthma DERs (*n* = 101, *P* = 0.076 Fisher’s exact test) overlapped with GR-binding sites identified in dexamethasone (DEX)-treated airway epithelial cells (Kadiyala et al., 2016), suggesting that glucocorticoid treatment was not a primary cause of epigenome reorganization in these subjects (Supplementary Figure S1B).

Although occurring primarily at intergenic sites genome-wide, asthma DERs were proximal to *n* = 3,062 genes and pathway analysis revealed that DER-associated genes contributed to epithelial (e.g., cellular adherens) and asthma-associated processes (e.g., TGF-β signaling) with up to 30% of all genes within these pathways exhibiting differential H3K27ac in asthma (range 19.9–30.1%, Figures 1C–E).

### Epithelial and Asthma-Associated Super-Enhancers Are Associated With Enzymes Involved in Lipid Metabolism

**FIGURE 2:**
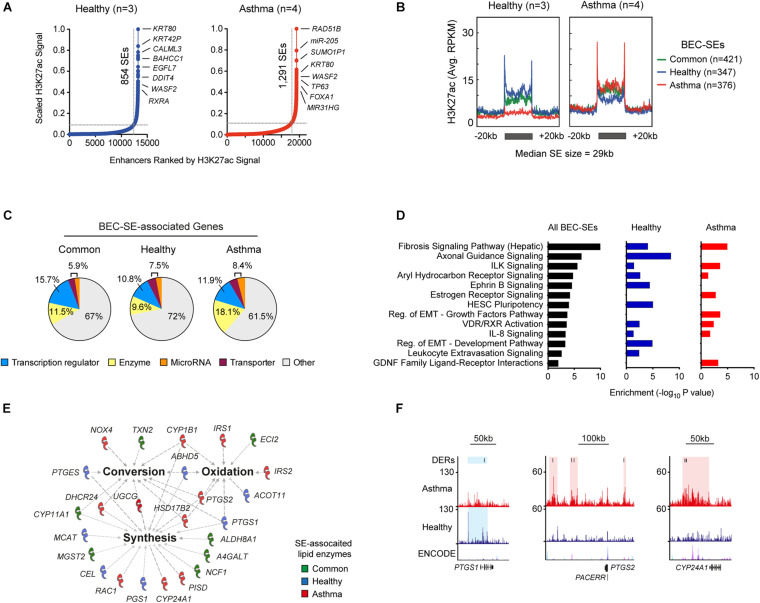
Identification of cell-specific and asthma-associated super-enhancers in the airway epithelium. **(A)** Ranking of H3K27ac ChIP-Seq signal from merged Healthy (blue, *n* = 3) and Asthma BECs (red, *n* = 4) identified super-enhancers in BECs (BEC-SEs) and associated genes. BEC-SEs were shared (e.g., *KRT80*) and unique to healthy (e.g., *CALML3*) or asthma (e.g., *RAD51B*). **(B)** “Meta gene” plots summarizing H3K27ac enrichment (RPKM) in healthy and asthma BECs across Common, Healthy, and Asthma-associated SEs. **(C)** Consistent with super-enhancers in other cells types, BEC-SE-associated genes included transcriptional regulators (blue). However, we also identified substantial numbers of BEC-SE-associated genes encoding enzymes (yellow). **(D)** Pathway analysis indicating that BEC-SE-associated genes were enriched in various pathways including fibrosis, axonal development, and those implicated in epithelial biology (e.g., EMT). **(E)** Lipid-associated processes (bold) and enzymes encompassed by BEC-SEs. **(F)** Genome tracks depict H3K27ac enrichment, DERs, and location of BEC-SEs across select lipid-associated enzymes.

Since we profiled an enhancer-associated histone modification, we sought to determine super-enhancer landscapes in BECs (SEs, Figure 2A). We identified and compared SEs from each volunteer revealing the presence of Common (airway cell-specific), Healthy, and Asthma-associated SEs (Supplementary Table S4). Plotting enrichment across BEC-SEs revealed dramatic changes in H3K27ac (Figure 2B and Supplementary Figure S1C), and 39.4% of SEs encompassed an asthma DERs. Plotting fold change further identified Asthma BECs exhibiting marked decreases in H3K27ac in Common and Healthy SEs and increased H3K27ac in Asthma SEs (Supplementary Figure S1D).

BEC-SE-associated genes encompassed a diverse range of biological processes including transcription regulators and enzymes (Figure 2C and Supplementary Table S4). Similar to DERs, BEC-SEs contributed to epithelial-associated pathways with Healthy and Asthma exhibiting differences across components of the same pathway (e.g., EMT development and growth factor signaling respectively, Figure 2D).

Because a greater proportion of asthma-SE-associated genes encoded enzymes than transcriptional regulators (18.15 vs. 11.9%, respectively), we focused on BEC-SE-associated enzymes and found they were enriched in metabolic processes, particularly the conversion, synthesis, and oxidation of lipids (Figure 2E and Supplementary Figure S1E). Further investigation revealed several lipid-associated enzymes implicated in asthma pathogenesis to be encompassed by asthma-SEs (e.g., *PTGS1*, *PTGS2*, *CYP24A1*, Figure 2F).

### Transcription Factors Associated With H3K27ac in Asthma

**FIGURE 3:**
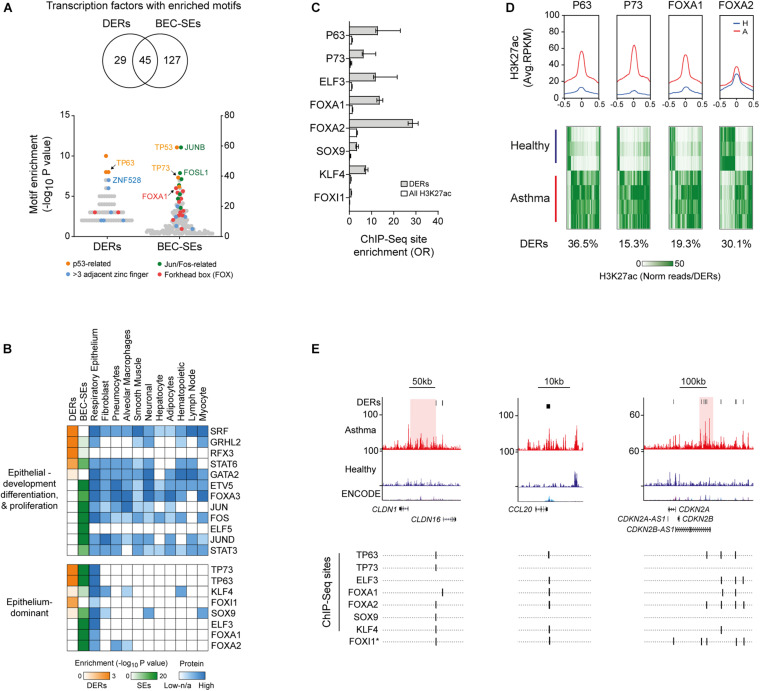
Identification of airway epithelium-dominant transcription factors (TFs) associated with H3K27ac in asthma. **(A)** Enrichment of TF motifs across asthma DERs and BEC-SEs. Examples of the most enriched and prevalent TF families are indicated. **(B)** Heat map depicting motif enrichment, protein expression, and tissue distribution of TFs enriched in asthma DERs and BEC-SEs, although representing most enriched TFs which were expressed across many tissue types. However, a subset of TFs was expressed almost exclusively in the respiratory epithelium (bottom panel). **(C)** Enrichment of DERs at epithelium-dominant TF-binding sites identified via ChIP-Seq. All enrichments in DERs and H3K27ac in BECs were significant (*P* < 0.0001, Fisher’s odds ratio ± 95% CI). **(D)** H3K27ac profiles of healthy (blue) and asthma (red) BECs at epithelium-dominant TF-binding sites identified via ChIP-Seq. Plots represent enrichment ± 0.5 kb around the binding site center. Heatmaps depict H3K27ac enrichment for each DER containing epithelium-dominant TF-binding sites across study volunteers. Proportion of total DERs with sites indicated below. **(E)** H3K27ac enrichment and ChIP-Seq-validated or predicted (^∗^) epithelium-dominant TF-binding sites across epithelial, cell recruitment, and cell cycle-associated genes. *CLDN1* and *CDKN2B-AS1* are encompassed by asthma-associated SEs (red).

We next identified enrichment of TF-binding motifs in DERs and BEC-SEs, predominately from members of the p53-related, Jun, Fos, and forkhead box TF-families (Figure 3A). While most TFs identified had protein expression across many tissue types, a subset of TFs with protein expression predominately in the respiratory epithelium were identified (e.g., TP63, Figure 3B).

We focused on Epithelium-dominant TFs and found those encompassed by BEC-SEs (Supplementary Figure S1F) have been previously implicated in lung development (*FOXA1*) (Besnard et al., 2005) and asthma, nasal polyposis, and mucociliary development (*TP63*, *TP73*) (Hackett et al., 2009; Li et al., 2009; Warner et al., 2013; Nemajerova et al., 2016). We found DERs were enriched at bona-fide epithelial TF sites as determined by ChIP-Seq (Figure 3C). Profiling revealed Asthma BECs on average exhibited more H3K27ac at epithelial-TF ChIP-Seq sites, encompassing up to 36.5% of asthma DERs (Figure 3D). However, plotting H3K27ac enrichment across study volunteers indicated both gain and loss at Epithelium-dominant TF sites. Finally, we found Epithelium-dominant TF binding sites co-localized in DERs populating genes associated with epithelial integrity (e.g., *CLDN16*), inflammatory cell recruitment (e.g., *CCL20*), and cell cycle (e.g., CDKN2A, Figure 3E).

### Alterations to H3K27ac Are Related to Asthma-Associated Transcriptional Profiles

**FIGURE 4:**
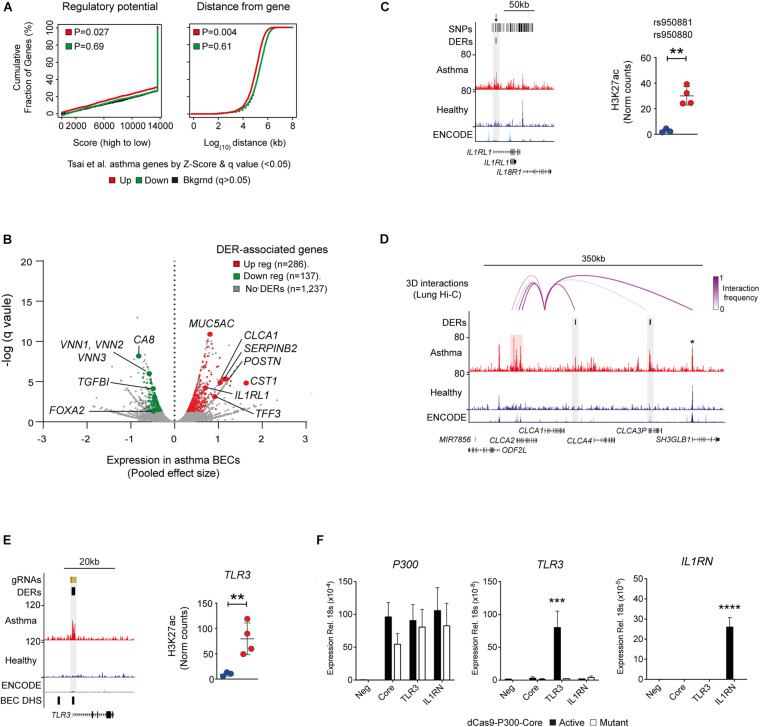
Relationship between H3K27ac and gene expression in asthma. **(A)** Plots generated by BETA analysis depicting the relationship between DERs and Asthma gene expression in airway epithelium identified by Tsai et al. (2018). Overall, gain in H3K27ac was associated with transcriptional activation (up regulated genes—red) and was closely related to genomic distance. **(B)** DER-associated genes and respective upregulation (red) and downregulation (green) in asthma identified by Tsai et al. (2018) (see Supplementary Table S5). **(C)** Genome tracks and dot plot depicting H3K27ac of DERs overlapping asthma SNPs at *IL1RL1* (orange lines and arrows, top track) in healthy (blue) and asthma (red) BECs. (^∗∗^*P* < 0.01, DiffBind). **(D)** Genome tracks depicting H3K27ac in BECs and long-range chromosomal interactions in lung tissue (top track) across the *CLCA1* locus. Although exhibiting minimal H3K27ac in BECs, *CLCA1* interacts with asthma DERs spanning ∼200 kb (purple arcs) including those near *CLCA3P* and an asthma-SE at *CLCA2* (highlighted in red). Note that no change in H3K27ac is evident at *SH3GLB1* (star). **(E)** Genome tracks depicting H3K27ac across the TLR3 location of DER-specific gRNAs (top track—orange lines). Regions of “open chromatin” identified in BECs are depicted in the bottom track (DNase hypersensitivity sites—DHS). Dot plots depicting H3K27ac enrichment of DER targeted by gRNAs across healthy (blue) and asthma volunteers (^∗∗^*P* < 0.01, DiffBind). **(F)** Using a CRISPR-based approach, acetylation (dCas9-P300-Core) was targeted to an asthma-DER at TLR3 using pooled guide RNAs. Increased P300 indicated successful delivery and expression of dCas9 constructs across all gRNA pools. However, TLR3 gene expression was selectively induced in a DER-specific gRNA and acetylation-dependent manner. A pool of non-DER-targeting gRNAs to IL1RN was used as control. Core—only P300 constructs transfected (*n* = 3, Mean ± SEM, ^∗∗∗^*P* < 0.001, ANOVA/Tukey multiple comparisons). ^*⁣*⁣**^p < 0.0001.

Since histone acetylation is associated with transcriptional activation, we investigated the relationship between H3K27ac and gene expression in asthma (Tsai et al., 2018). We determined that changes in H3K27ac were associated predominately with upregulated genes, and this relationship was more pronounced when genomic distance of DERs from differentially expressed genes was considered (Figure 4A). Genomic distance was also found to associate BEC-SE with gene expression in the asthma epithelium (*P* = 0.01—data not shown).

We found key asthma-associated genes including the T2-high signature genes of *CLCA1*, *POSTN*, and *SERPINB2* (Woodruff et al., 2007) as well as those not previously asthma-associated (e.g., *CST1* and Vanin genes—*VNN1–3*) to be potentially influenced by aberrant H3K27ac (Figure 4B). Interestingly, we found the DER at *IL1RL1* (also known as the IL33 receptor ST2) overlapped previously identified asthma-associated SNPs (Figure 4C).

While further investigation revealed no enrichment of asthma SNPs in DERs, BEC-SEs exhibited a near significant enrichment for asthma SNPs (Supplementary Figure S1G).

To further elucidate the relationship between genomic distance, DERs and gene expression (Figure 1C), we investigated if similar to asthma SNPs (Nicodemus-Johnson et al., 2016; Schmiedel et al., 2016); DERs and BEC-SEs are connected to DE genes via long-range chromosomal interactions. We utilized lung Hi-C data and found that DERs and BEC-SE exhibited strong 3D interactions, particularly for regions encompassing *ARGN*, *SMAD11*, and *AQP7P1* and *LOC442421*, respectively (Supplementary Figure S1H).

We then focused on the locus of *CLCA1* (Woodruff et al., 2009) as it exhibited the highest interaction frequency of all T2-high signature genes. We observed interactions between *CLCA1* and asthma DERs located downstream and an asthma-SE located upstream encompassing the *CLCA2* transcriptional start site (Figure 4D). Remarkably, there was minimal H3K27ac enrichment across the *CLCA1* gene in either healthy or asthma BECs suggesting that transcriptional control of *CLCA1* requires interactions with distal regulatory elements.

To clarify the relationship between the epigenome and transcriptome in asthma, we employed a CRISPR-Cas9-based approach to recapitulate the epigenome of asthma BECs in human embryonic kidney (HEK293T) cells *in vitro* (Lavender et al., 2018). HEKs were chosen because they do not express some of the genes upregulated in asthma (*CLCA1*, *POSTN*, *SERPINB2*) and as such mirror expression of healthy BECs. We identified gRNAs that successfully targeted acetylation to asthma DERs at TLR3 and found that gene expression could be induced in an acetylation-specific manner. Use of a mutant dCas9- had no inductive effect upon transcription. These data provide a framework to apply epigenome editing in more relevant cell types to clarify the relationship between asthma-associated epigenome and transcriptional profiles (Figures 4E,F).

## Discussion

Our study sought to pilot genome-wide profiling of an enhancer-associated histone modification in BECs and establish evidence for differences in asthmatic volunteers compared to healthy counterparts. Differential H3K27ac enrichment in asthma BECs clustered predominately around epithelial-associated genes/pathways (Figure 1C). While our small sample size does not cover the heterogeneity and spectrum of asthma severity, our data is consistent with studies of other epigenomic mechanisms (Nicodemus-Johnson et al., 2016; Yang et al., 2017) and adds to growing evidence that chronic inflammatory airway disease is characterized by distinct changes to the epigenome.

By profiling an enhancer-associated histone modification, we could identify initial evidence of airway epithelial cell- and asthma-associated SEs (Figure 2). While validation in a larger cohort is needed, we found that asthma-SEs encompassed transcription factors (*TP63*) (Hackett et al., 2009) and enzymes important in lipid metabolism (e.g., *PTGS1*, *PTGS2*, Supplementary Table S4; Juncadella et al., 2013). Given that lipid mediators are key components of the inflammatory response and targets of current (e.g., montelukast) and novel asthma therapies (e.g., fevipiprant) (Gonem et al., 2016), further investigations are required to determine the consequence of epigenome perturbations surrounding enzymes responsible for mediator synthesis.

We sought to identify the molecular components associated with changes in H3K27ac in asthma BECs and identified a transcription factor signature that was enriched for factors known to drive airway epithelium lineage determination (e.g., FOX and P53 family members). We found that a number of TFs were encompassed by BEC-SEs including TP63, a well-defined marker of basal cells, and TP73, a key player in mucociliary development (Supplementary Figure S1E) and observed enrichment for FOXI1 sites in asthma DERs, a TF shown to characterize the recently identified ionocytes (Montoro et al., 2018; Plasschaert et al., 2018). We postulate that the TFs identified in our analyses reflect the “master TFs” of discreet cell populations comprising the airway epithelium and how this admixture may differ in people with asthma. Consequently, genome-wide profiling studies are needed to establish whether the TFs enriched in Asthma DERs represent the drivers initiating and exploiting changes we observed in the histone landscape of asthma BECs.

We found that the gain and loss of H3K27ac was associated with concurrent changes in asthma-associated gene expression (Tsai et al., 2018) including T2-high signature genes and transcription factors (e.g., FOXA2, Figure 4B). However, because H3K27ac is enriched predominately in intergenic (i.e., non-coding) regions (Supplementary Figure S1A), it is becoming apparent that the complexities between epigenetics and transcriptome may be clarified by taking higher-order chromatin architecture into account. When we integrated 3D architecture data into our own analyses, we observed long-range interactions connected DERs across the CLCA1 loci (Figure 4D). Our observations add to growing evidence that 3D architecture link epigenetics, genetics, and the transcriptome in asthma (Nicodemus-Johnson et al., 2016; Schmiedel et al., 2016).

Since proximity does not necessarily define the preferred regulatory target, an ongoing challenge of genome-wide epigenetic analysis is the determination of which enhancers/regulatory domains influence activity of promoters. Studies by Klann et al. (2017) have indicated that the ability to manipulate the epigenome using dCas9 approaches can be cell-type specific and that when nucleosomes in a target region in a population of cells are already fully or partially acetylated, dCas9-based epigenome editing will only produce a modest effect due to lack of substrate availability. Furthermore, the combination of other regulatory domains/factors or stimuli may be needed to define expression capability. Indeed, we found that targeting of DERs within asthma-SEs at *SERPINB2* did not result in induction of gene expression (Supplementary Figure S1I). Regardless, dCas9-based epigenome editing provides a tremendous tool to help clarify the functional consequences of asthma-associated changes in the epigenome (Lavender et al., 2018).

It remains to be determined what the trigger(s) mediating the changes in the epigenomic landscape we identified in asthma is/are. Future longitudinal studies involving larger sample numbers, covering the spectrum of asthma severities and including matched transcriptomic and epigenomic datasets, are needed to determine when the epigenome is altered in asthma and if there are key “epigenomic windows” of disease development that might benefit through therapeutic intervention.

## Methods

### Patient Recruitment

Volunteers with and without asthma were recruited as previously described (Dhariwal et al., 2017). None were smokers or had had asthma exacerbations or any respiratory tract infections in the preceding 6 weeks (Supplementary Table S1). Asthma was defined as a physician’s diagnosis of asthma, and volunteers with asthma had airway hyper-responsiveness (provocative concentration (PC_20_) of histamine required to reduce FEV_1_ by = 20% of = 8 μg/mL, an Asthma Control Questionnaire (Juniper et al., 2006) score > 0.75, and were on treatment with inhaled corticosteroids (ICSs) or a combination inhaler (long-acting β agonist + ICS). Healthy controls had a PC_20_ histamine > 8 μg/mL. All volunteers with asthma were atopic and all healthy controls non-atopic as determined by skin prick testing (at least one positive skin prick test to a panel of 10 aeroallergens, including grass).

### Primary Bronchial Epithelial Cell (BEC) Culture

Bronchial brushings were obtained from volunteers by fiber-optic bronchoscopy using a Keymed BF260 bronchoscope (Olympus, Essex, United Kingdom) and 5 mm sheathed endobronchial brushes (Olympus BC-202D-5010) in accordance with British Thoracic Society guidelines (British Thoracic Society Bronchoscopy Guidelines Committee, 2001). Freshly brushed BECs were removed from brushes by agitation and seeded into a supplemented bronchial epithelial growth medium (BEBM, Lonza) in a T25 flask. Cell culture was performed as described previously (Edwards et al., 2013) and seeded at passage 2 onto 10 mm culture dishes (Primaria, Corning) and cultured until 80% confluence.

### Chromatin Immunoprecipitation

BECs were trypsinized and lysed in hypotonic/mild detergent buffer (15 Mm Tris–HCL pH 7.5, 60 mM KCL, 15 mM NaCL, 5 mM MgCl_2_ 0.1 mM EGTA, 5 mM DTT, 0.4% IGEPAL-CA), and nuclei were isolated through sucrose density gradient (0.3/1.2 M sucrose, 20,000 g, 20 min, 4°C). Viability of nuclei was assessed via trypan blue staining. Nuclei were treated with micrococcal nuclease (10U, NEB) for 10 min at 37°C and repelleted and supernatant containing mononucleosomes stored at 4°C. Mononucleosomal fractions were incubated with 4 μg of anti-H3K27ac antibody (Abcam, Ab4729) and 25 μL Protein G dynabeads (Life Technologies) in modified RIPA buffer overnight at 4°C. Bound complexes were washed and eluted and ChIP DNA extracted using phenol:chloroform/ethanol precipitation.

### Sequencing and Mapping

Libraries were prepared using half total volume of eluted ChIP DNA and NEBNext^®^ DNA Library Prep Master Mix Set and Multiplex Oligos for Illumina^®^ (New England Biolabs). Library quality was assessed using Bioanalyzer 2100 High Sensitivity DNA Gels (Agilent). Libraries were subject to 50-bp single end read sequencing on HighSeq 2500 (Illumina) in rapid run format, and reads were aligned to Human genome hg19 using Bowtie2 (Galaxy v2.2.6) Afgan et al., 2016; Supplementary Table S2). ENCODE and BEC-associated blacklist regions (including chr*N*, chr*Un*, and chrM) were subtracted from each BAM file prior to downstream analysis using the *intersect -v* function in bedtools (Galaxy v2.1.0). For visualization, Healthy (*n* = 3) and Asthma (*n* = 4) BAM files were merged using samtools (Galaxy), Input BAMs subtracted (*-bamCompare*), and BigWig’s generated and normalized to reads per kilobase per million mapped reads (RPKM, *-bamCoverage*, deepTools) (Ramírez et al., 2014).

### Differential Analysis and Super-Enhancer Identification

Peaks were called for merged Healthy, Asthma, and individual volunteer BAM files using MACS2 (*p* < 5e-4, Galaxy v2.1, Supplementary Table S2, S4). Differentially enriched regions (DERs) between Asthma (*n* = 4) and Healthy BECs (*n* = 3) were identified from a consensus set of peaks (*n* = 42,168) using DiffBind (Stark and Brown, 2011) (*Summits* = *500*, *FDR Threshold* = *0.05*, Supplementary Table S3). Genomic distribution of DERs was determined using CEAS (CISTROME v1.0.0) and DERs annotated using *ROSE_geneMapper.py* (see below) and HOMER (Heinz et al., 2010).

Super-enhancers (SEs) as defined by Hnisz et al. (2013) were identified and annotated using the Rank Ordering of Super Enhancers algorithm (*ROSE_main.py*, *-t 2000*, and *ROSE_geneMapper.py*, *searchwindow* = *default*, respectively, Supplementary Table S4) (Hnisz et al., 2013). Individual volunteer SEs were compared using *merge-o distinct, count* function in bedtools to identify SE categories. Briefly, to strike a balance between small volunteer numbers and disease heterogeneity, Common SEs were defined as those being present in *n* = 4/7 volunteers, Healthy SEs; *n* = 2/3 healthy volunteers, and Asthma SEs; *n* = 3/4 asthma volunteers and no further overlap with any other SE category. BEC-SEs were intersected with Asthma DERs and coverage of DERs across BEC-SEs determined using the *intersect* and *coverageBed* options in bedtools.

### Transcription Factor Motif Enrichment

Motif enrichment in DERs (*n* = 4,321) and peaks within BEC-SEs (*n* = 12,457) was conducted using HOMER (*findMotifsGenome.pl -mknown* = *1HOCOMOCOv11 core HUMAN mono homer format 0.001.motif*) and options for DERs (*-size given, -h -bg* = *all test peaks*) and BEC-SEs (*-size 500*) (Heinz et al., 2010; Kulakovskiy et al., 2016). We then selected enriched TFs (*P* < 0.05) associated with epithelial processes via Ingenuity^®^ (see below) and/or had motifs enriched in BEC-SEs and/or had protein expressed in the respiratory epithelium for further analysis (*n* = 201, data not shown). Protein expression and tissue specificity of TFs were determined via Human Protein Atlas (normal tissue data v.16.1) (Uhlén et al., 2015). Heatmaps were produced using Morpheus. Published TF ChIP-Seq datasets were downloaded from ChIP-Atlas (Oki et al., 2018) and merged to create a consensus list of bona-fide TF-binding sites and intersected with DERs using bedtools. HOMER (*annotatePeaks.pl -m* = *HOCOMOCOv11 core HUMAN mono homer format 0.001.motif*) was used to predict binding sites for those TFs with no ChIP-Seq data available. Binding site enrichment was determined using Fisher’s exact test (as above) and the consensus peak set identified in DiffBind analysis (*n* = 42,168 peaks) used as background. H3K37ac enrichment profiles ± 0.55 kb around the intersecting DER center were plotted using *plotProfile* in deepTools.

### Pathway Analysis

All pathway and biological process enrichment analyses and SE-associated gene categorizations were conducted using Ingenuity^®^ Pathway Analysis Software (QIAGEN).

### Asthma SNP Enrichment

DERs and BEC-SEs were intersected with Asthma SNPs compiled by Seumois et al. (2014) and enrichment determined with Fisher’s exact test in GraphPad Prism v.8.1.

### Transcriptomics

We investigated gene expression using meta-analysis of the asthma airway epithelium, performed by Tsai et al. (2018). Relationships between differential gene expression and asthma DERs were determined using BETA (Cistrome v1.0.0, *geneID* = *Refseq, genome* = *hg19, peaks* = *30,000, TSS distance* = *250,000, CTCF* = *True, significance* = *0.05*) (Wang et al., 2013). Briefly, by assigning a rank number based on the distance between asthma DERs and DE genes (*x*-axis) and plotting them vs. the proportion of genes with ranks at or better than the *x*-axis value (*y*-axis), BETA identifies the potential activating/repressing function of asthma DERs on gene expression.

### 3D Chromosomal Interactions

To investigate relationships between long-range interactions and DERs, chromosomal interaction data (Hi-C) was accessed via 3D Interaction Viewer and Database (Yang et al., 2018). Since no chromosomal interaction data is available for normal airway epithelial cells, we accessed datasets from whole lung tissue and intersected it with DERs and BEC-SEs using bedtools. For plotting, interactions around *CLCA1* search parameters were *Bait* = *CLCA1* TSS, *Interaction range* = *500 kb*, and *TAD* = *TopDom (w* = *20)*.

### Targeting Acetylation to Asthma DERs

Plasmids encoding active and mutant forms of a deactivated-Cas9-histone acetyltransferase P300 fusion protein (pcDNA-dCas9-p300 Core, pcDNA-dCas9-p300 Core-D1399Y) and guide RNA only expression vector (phU6-gRNA) were gifts from Charles Gersbach (Hilton et al., 2015) (Addgene #61357, #61358, and #53188, respectively). DER-specific gRNAs were designed by submitting DER coordinates or sequence to the CRISPOR^1^ and Breaking-Cas (Oliveros et al., 2016) servers, respectively, and selected based on overlap with BEC DNAse hypersensitivity sites (ENCODE). Control gRNAs to IL1RN were described previously (Perez-Pinera et al., 2013). gRNAs were annealed and cloned into phU6-gRNA via *Bbs*I restriction sites (NEB #R0539S). All plasmids were transformed into OneShot^®^TOP10 competent cells (Thermo Fisher Scientific), cultured overnight, and extracted using Endo Free^®^ Plasmid Maxi or QIAprep^®^ Spin Miniprep (gRNAs) kits (QIAGEN). gRNAs sequence and locations are listed in Supplementary Table S5.

### Cell Culture and Transfections

HEK-293T cells were maintained in DMEM media supplemented with 10% fetal calf serum and penicillin/streptomycin at 37°C 5% CO_2_. 2 × 10^5^ cells were reverse transfected with 1 μg of dCas9-P300 constructs and 125 ng of equimolar pooled or individual gRNAs using Lipofectamine 3000 according to the manufacturer’s instructions (Thermo Fisher Scientific). Cells were harvested at 48 h and RNA extracted (RNAeasy^®^ Mini Kit, QIAGEN). Gene expression was determined via Taqman qPCR assays (Supplementary Table S5) and run on the Applied Biosystems ViiA^TM^ 7 Real Time PCR system (Thermo Fisher Scientific).

### Glucocorticoid Receptor ChIP-Seq

FASTQ files from dataset GSE79803 (Kadiyala et al., 2016) was downloaded from SRA (SRP072707) and processed as outlined above. DERs were intersected with dexamethasone (DEX)-responsive GR sites as described by Kadiyala et al. (2016) using bedtools and GR enrichment profiles ± 5 kb around intersecting sites plotted with deepTools.

## Data Availability Statement

The datasets presented in this study can be found in online repositories. The names of the repository/repositories and accession number(s) can be found below: https://www.ncbi.nlm.nih.gov/genbank/.

## Ethics Statement

This study was approved by the London Bridge Research Ethics Committee (reference 10/LO/1278) and was carried out in accordance with the Declaration of Helsinki and Good Clinical Practice guidelines. Informed consent was obtained from all subjects prior to their participation. The patients/participants provided their written informed consent to participate in this study.

## Author Contributions

JD performed the clinical aspects of the study. SJ supervised the clinical aspects of the study. ME performed the clinical sample processing and culture. PM, AK, and JW performed the ChIP Experiments. PM and PL analyzed the ChIP-Seq data. PM and MK performed the dCas9 CRISPR work. PL, DC, PM, JS, RS, AO, ME, and SJ conceived and designed the study. All authors contributed to the article and approved the submitted version.

## Conflict of Interest

JS and AO were employed by the company GlaxoSmithKline. The remaining authors declare that the research was conducted in the absence of any commercial or financial relationships that could be construed as a potential conflict of interest.

## Footnotes

1 http://crispor.tefor.net

## Supplementary Material

Supplementary Figure 1 **(A)** Distribution of Asthma DERs across genomic features. TSS – transcriptional start site, UTR – un-translated region. **(B)** Overlap and enrichment between asthma DERs and dexamethasone (DEX)-responsive glucocorticoid receptor (GR) binding sites identified in the BEAS-2B airway epithelial cell line. Fishers odds ratio ± 95% CI. **(C)** Heatmap depicting H3K27ac enrichment for each volunteer across BEC-SE categories. **(D)** Plot depicting log fold change (FC) of all asthma DERs located within BEC-SEs. Asthma BECs exhibited marked changes in H3K27ac across airway cell-specific (i.e., common, green) and healthy- (blue) or asthma- (red) SEs (median ± max-min values, ^∗∗∗^*P* < 0.001, ANOVA/Tukeys multiple comparisons). **(E)** Pathway analysis indicating that BEC-SE-associated enzymes were enriched in various metabolic processes, particularly those associated with lipids. **(F)** Genome tracks depicting H3K27ac across loci encoding epithelium-dominant TFs encompassed by BEC-SEs and asthma DERs. Asthma = red, healthy = blue. **(G)** Enrichment of asthma SNPs across DERs and other features of H3K27ac in BECs (Fishers odds ratio ± 95% CI). **(H)** Plot depicting average 3D interaction frequency of features identified in this study across whole lung HiC data. T2-Signature genes of *CLCA1*, *POSTN*, and *SERPINB2* are included separately as reference. **(I)** Genome tracks depicting H3K27ac in BECs across the SERPINB2. Acetylation (dCas9-P300-Core) was targeted to the TSS (pool 1) of SERPINB2 and upstream within an asthma-associated SE (pools 2–4) using gRNAs (yellow boxes top tracks). No induction of gene expression was observed when targeting distal enhancer elements. Guide RNAs to IL1RN used as controls. Core—only P300 constructs transfected (*n* = 3, Mean ± SEM, ^∗∗^*P* < 0.01, ANOVA/Tukeys multiple comparisons).

Supplementary Table 1 Clinical characteristics of study volunteers. ACQ, asthma control questionnaire; FEV_1_, forced expiratory volume in 1 s; PC_20_, provocative concentration of histamine to cause a 20% decrease in FEV_1_; SPT, skin prick test.

Supplementary Table 2 Statistics for H3K27ac ChIP-Seq data and analysis.

Supplementary Table 3 Differentially enriched regions (DERs) of H3K27ac in Asthma BECs. Table lists all DERs FDR *P* < 0.05, associated genes and H3K27ac enrichment across all study volunteers. Additional annotations by HOMER are included.

Supplementary Table 4 Super-enhancers of the airway epithelium. Table lists location, associated genes and coverage of asthma DERs for all common, healthy and asthma associated-SEs.

Supplementary Table 5 Influence of H3K27ac on asthma gene expression. Asthma-associated genes identified by Tsai et al. (2018) are ranked by regulatory potential as determined by BETA analysis. Number of DERs per gene are additionally included to association analysis conducted by Tsai et al. (2018).

Supplementary Table 6 Sequence and location of DER-specific and control guide RNAs.

## Abbreviations

3D: three dimensions
BECs: bronchial epithelial cells
CGI’s: CpG Islands
ChIP-Seq: chromatin-immunoprecipitation coupled with high-through-put sequencing
CRISPR-Cas9: clustered regularly interspaced short palindromic repeats
DERs: differentially enriched regions
DEX: dexamethasone
ENCODE: Encyclopedia of DNA Elements consortia
gRNA: guide RNA
H3K27ac: histone H3 Lys27 acetylation
H3K4me2: histone H3 Lys4 dimethylation
kb: kilobase
RPKM: reads per kilobase per million mapped reads
SEs: super enhancers
SNPs: single-nucleotide polymorphisms
T2: type 2 airway inflammation
TFs: transcription factors
TSSs: transcriptional start sites.
