# Supplementary figures and images for: Profiling of H3K27Ac Reveals the Influence of Asthma on the Epigenome of the Airway Epithelium
Source: Front Genet. 2020 Dec 10;11:585746. doi: 10.3389/fgene.2020.585746 (PMC7758344; doi:10.3389/fgene.2020.585746)

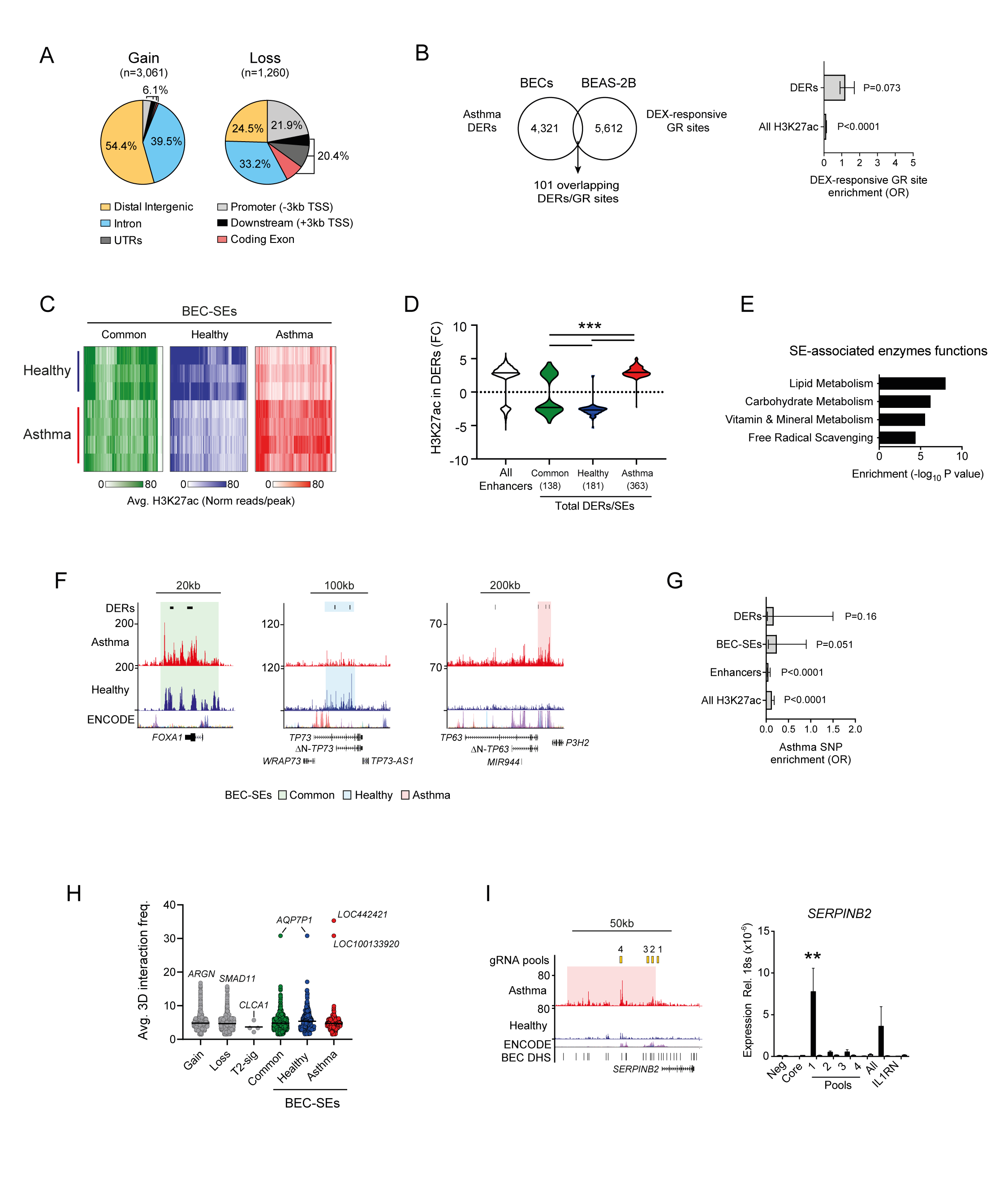

Supplement: Supplementary Figure 1 — (A) Distribution of Asthma DERs across genomic features. TSS – transcriptional start site, UTR – un-translated region. (B) Overlap and enrichment between asthma DERs and dexamethasone (DEX)-responsive glucocorticoid receptor (GR) binding sites identified in the BEAS-2B airway epithelial cell line. Fishers odds ratio ± 95% CI. (C) Heatmap depicting H3K27ac enrichment for each volunteer across BEC-SE categories. (D) Plot depicting log fold change (FC) of all asthma DERs located within BEC-SEs. Asthma BECs exhibited marked changes in H3K27ac across airway cell-specific (i.e., common, green) and healthy- (blue) or asthma- (red) SEs (median ± max-min values, ∗∗∗P < 0.001, ANOVA/Tukeys multiple comparisons). (E) Pathway analysis indicating that BEC-SE-associated enzymes were enriched in various metabolic processes, particularly those associated with lipids. (F) Genome tracks depicting H3K27ac across loci encoding epithelium-dominant TFs encompassed by BEC-SEs and asthma DERs. Asthma = red, healthy = blue. (G) Enrichment of asthma SNPs across DERs and other features of H3K27ac in BECs (Fishers odds ratio ± 95% CI). (H) Plot depicting average 3D interaction frequency of features identified in this study across whole lung HiC data. T2-Signature genes of CLCA1, POSTN, and SERPINB2 are included separately as reference. (I) Genome tracks depicting H3K27ac in BECs across the SERPINB2. Acetylation (dCas9-P300-Core) was targeted to the TSS (pool 1) of SERPINB2 and upstream within an asthma-associated SE (pools 2–4) using gRNAs (yellow boxes top tracks). No induction of gene expression was observed when targeting distal enhancer elements. Guide RNAs to IL1RN used as controls. Core—only P300 constructs transfected (n = 3, Mean ± SEM, ∗∗P < 0.01, ANOVA/Tukeys multiple comparisons). [file Image_1.TIF]
